# Replicating medication trend studies using ad hoc information extraction in a clinical data warehouse

**DOI:** 10.1186/s12911-018-0729-0

**Published:** 2019-01-18

**Authors:** Georg Dietrich, Jonathan Krebs, Leon Liman, Georg Fette, Maximilian Ertl, Mathias Kaspar, Stefan Störk, Frank Puppe

**Affiliations:** 1 Computer Science, Unviversity of Würzburg, Am Hubland, Würzburg, 97074 Germany; 2 Comprehensive Heart Failure Center, University and University Hospital Hospital of Würzburg, Am Schwarzenberg 15, Würzburg, 97078 Germany; 3 Service Center Medical Informatics, University Hospital of Würzburg, Schweinfurter Strasse 4, Würzburg, 97078 Germany

**Keywords:** Data warehouse, Medication extraction, Information extraction

## Abstract

**Background:**

Medication trend studies show the changes of medication over the years and may be replicated using a clinical Data Warehouse (CDW). Even nowadays, a lot of the patient information, like medication data, in the EHR is stored in the format of free text. As the conventional approach of information extraction (IE) demands a high developmental effort, we used ad hoc IE instead. This technique queries information and extracts it on the fly from texts contained in the CDW.

**Methods:**

We present a generalizable approach of ad hoc IE for pharmacotherapy (medications and their daily dosage) presented in hospital discharge letters. We added import and query features to the CDW system, like error tolerant queries to deal with misspellings and proximity search for the extraction of the daily dosage. During the data integration process in the CDW, negated, historical and non-patient context data are filtered. For the replication studies, we used a drug list grouped by ATC (Anatomical Therapeutic Chemical Classification System) codes as input for queries to the CDW.

**Results:**

We achieve an F1 score of 0.983 (precision 0.997, recall 0.970) for extracting medication from discharge letters and an F1 score of 0.974 (precision 0.977, recall 0.972) for extracting the dosage. We replicated three published medical trend studies for hypertension, atrial fibrillation and chronic kidney disease. Overall, 93% of the main findings could be replicated, 68% of sub-findings, and 75% of all findings. One study could be completely replicated with all main and sub-findings.

**Conclusion:**

A novel approach for ad hoc IE is presented. It is very suitable for basic medical texts like discharge letters and finding reports. Ad hoc IE is by definition more limited than conventional IE and does not claim to replace it, but it substantially exceeds the search capabilities of many CDWs and it is convenient to conduct replication studies fast and with high quality.

## Background

Reliable information on the use of medication in a hospital and its changes over time is of great importance for many acute and chronic diseases – from a hospital, patient and payor perspective. This is reflected by many studies reporting medication trends: e.g. attention deficit hyperactivity disorder (ADHD) [1], atrial fibrillation (AF) (US [2], Denmark [3, 4]), chronic kidney disease (CKD) [5, 6], rheumatoid disease [7] or hypertension (HT) [8] (England [9], France [10], Germany [11], Sweden [12], US [13, 14]).

However, medical research (like many other disciplines) is affected by the so called replication crisis, addressed in an article in 2012 reporting that only 11% of the pre-clinical cancer studies could be replicated [15]. The Nature Journal conducted a survey of 1500 scientists in 2016, in which 70% of them stated that they had failed to reproduce another scientist’s experiment [16].

The ability to reproduce findings reported in a clinical study is a cornerstone of scientific progress. Replication of medication trend studies can be performed using a CDW, which is an important, albeit little exploited and published use case.

CDWs can deal with structured data very well. Unfortunately, a lot of the patient information in the electronic health record (EHR) is still stored in free text. E.g. Jensen et al. retrieved on average 146 unstructured text documents for each patient from EHR of their hospital for their study [17]. Medication, too, is usually documented as free text within the discharge letter. As a solution, advanced CDW systems offer a query language that can extract data from free text (e.g. in [18]).

The conventional approach is to perform information extraction (IE) in the ETL^1^ process. A well-known system for IE of medication is MedEx [19]. Beside other rule based-systems like [20], hybrid systems exist using machine learning techniques [21]. A good overview on IE from free text is given by Wang et al. [22].

Rule based systems require a high volume of handcrafted rules and learning systems need a large amount of manually labeled training data. Either way, a lot of expert work is necessary. Besides high developmental efforts, another disadvantage of conventional IE is its slow promptness and non-adaptability by users [18].

A novel way to retrieve information from plain text is ad hoc IE. Ad hoc IE is described as extracting the existence of any concepts (e.g. chronic kidney disease) or any numbers, like the left ventricular ejection fraction (LVEF) value, from textual sources in real-time. The Boolean ad hoc IE queries the existence (yes/no) of a medical concept. A medical concept is a named entity that may have a feature/property or a numeric value. Examples of Boolean concepts are single findings or assessments (e.g. moderate mitral insufficiency, severe aortic stenosis), drugs (e.g. Aspirin, beta blocker) or diagnoses (e.g. appendicitis, myocardial infarction). Numeric IE extracts the value as number of a numerical concept. That could be for example the value of a laboratory finding (e.g. cholesterol, glucose, LEVF) or a derived values/indexes (e.g. BMI, age). A numerical condition can be defined optionally, like LVEF <45, matching all mentions of LVEF with a value lower than 45. In some finding reports, the exact value of a concept is not given but there is a formulation indicating an interval or an inequality of a value (e.g. “LVEF lower than 45"). These statements can be queried in conjunction with numeric ad hoc IE exploiting both qualitative and quantitative information from textual reports e.g. for checking inclusion or exclusion criteria of studies. In addition to count queries, which only asses the presence of a concept or the validity of constraints (e.g. BMI >25), the actual values can also be returned for further processing.

This technique showed good results and requires little developmental effort, since the text is indexed efficiently and can be queried with powerful features [18].

## Objectives

This work introduces ad hoc IE for medication and their daily dosage from hospital discharge letters. We present and evaluate query features for a CDW. As an example of use, we show medication trend estimations. Therefore we replicate existing studies from the literature in a large CDW of the University Hospital of Würzburg using ad hoc IE. The results will be compared with the corresponding published data describing similarities and differences.

## Methods

The developmental steps included extensions and features for the data integration process and the development of new data query tools. For study replication, the drug names had to be acquired and transformed.

### CDW system design

We implemented our features in the PaDaWaN CDW [23], which uses the full-text-search engine Apache Solr^2^ as storage engine, based on the index library Apache Lucene^3^. The PaDaWaN-CDW contains both, unstructured text data and structured data, including core data (e.g. age, sex etc.), coded data (e.g. ICD10 and OPS etc.) and numerous other types of information of the clinical information system (CIS) (e.g. lab data) [18]. The data integration process of the PaDaWaN-system contains analyzers for the respective data types. At the end of the pipeline, all values are stored in the Lucene index and can be queried from physicians in the PaDaWaN Web GUI [23]. We modified and extended generic tools for text analysis in the import pipeline (see below). We also added new query features to the framework, which can be used in the front end GUI during runtime.

### Data integration development

#### Lexical analysis

**Fig. 1:**
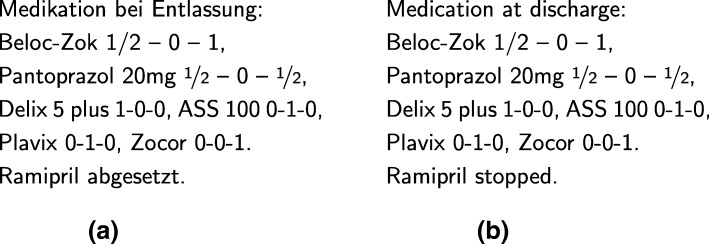
Example of a medication section of a hospital discharge letter. **a** German. **b** English

The text analysis tool for discharge letters splits the text into sections like diagnoses, medications, and laboratory values. Figure 1 shows an example for a medication section. We added a sentence splitter for medication extraction that separates the individual medication instructions from each other. Furthermore, we deactivated the stemmer because the word endings of the medications should not be touched. Finally, a custom tokenizer ensures that the quantity, strength and dosage information of the medication instructions are correctly decomposed. Table 1 shows an example of the lexical analysis.

**Table 1:** Lexical analysis of the medication section in the discharge letter

Text	Sentences	Tokens
Delix 10mg 1-0-0, Belok zok 1/2-0-0, Mono-Mack 20 1-1-0	Delix 10mg 1-0-0	Delix, 10, mg, 1, 0, 0
	Belok zok 1/2-0-0	Belok, zok, 1/2, 0, 0
	Mono-Mack 20 1-1-0	Mono, Mack, 20, 1, 1, 0

#### Context of information

The context of information in a discharge letter is an important topic. Many pieces of information are negated [24] (e.g. “no fever”, “dizziness is denied”) or they relate to other persons (e.g. within the context of family history). Some information like medications within in the discharge letter have a temporal context and may not be valid any longer (e.g. medication might have been stopped at hospital entry or during hospitalization, like Ramipril in Fig. 1). Depending on the application or evaluation, different types of information are relevant or must be excluded. In most cases, physicians are interested in the confirmed and current findings of a patient.

The PaDaWaN data integration process already identifies negations in the texts with an extended version of the NegEx-algorithm [25]. These negations can be excluded in the GUI for certain queries like medication extraction [18]. We extended this NegEx-version to a ConText [26] implementation. This algorithm handles not only negations but also the context of an information. It is implemented using Apache UIMA^4^. Furthermore, we added several trigger tokens for the patient history.^5^ Using these modifications, the non-currently used drugs are excluded from the text. The remaining, relevant medications remain retrievable at runtime by user queries.

### Text query features

#### Spelling error tolerant query

PaDaWaN already contains several text query features like token, phrase and regular expression queries. Since medical reports are often manually entered, some names of medications are misspelled. For such typos we added a spelling error tolerant query feature that makes use of the Damerau-Levenshtein distance. It is a string metric for measuring the edit distance between two sequences and can thus be employed to assess how much two medication names differ. The distance measures includes a transposition operation (transposition of two adjacent characters) in addition to three edit operations, i.e. insertion, deletion, and substitution [27]. Table 2 shows selected examples of misspellings and their Damerau–Levenshtein distance to the product name.

**Table 2:** Examples of misspelled medication names and their Damerau–Levenshtein distance

Product name	Misspells	Distance	Operation
Ibuhexal	Ibohexal	1	Substitution
Cordarex	Kordarex	1	Substitution
Warfarin	Wafarin	1	Snsertion
Euphylong	Euphyllong	1	Deletion
Repaglinid	Repagilnid	1	Transposition
Ramipril	Rampiril	1	Transposition
Repaglinid	Repagilid	2	Transposition, insertion

#### Dose extraction with proximity search

Although most medication trend studies only consider the use of a drug, we also strived to extract the daily dosage of the medication. This requires two pieces of information: the strength and the cumulative daily amount of the drug. The strength is given in digits with a standard unit (usually milligrams or micrograms) with the drug name. The dosing interval is usually coded by a number-hyphen notation like 1/2-0-1/2. The numbers represent the units that must be taken in the morning, at noon and in the evening. A optional fourth digit refers to the number before going to bed. The daily dose is obtained by adding these three or four numbers and then multiplying by the strength. We added a feature that makes it easier to query the daily dose. The proximity query searches the given tokens next to each other. The order of these tokens is irrelevant. Proximity queries do not match across sentence boundaries. Since each medication instruction is provided in a segmented fashion as a single sentence during the import, proximity queries do not match dosage information of other medications. Table 3 shows an example of how a daily dose can be extracted. The corresponding request is displayed as well as matching and not matching text snippets. With this technique, queries can be made for the different drug strengths and daily dosages.

**Table 3:** Example for promximity searches to query the daily dose of a medication instruction

Query	Expanded query	Matching	Not matching
Delix 5 mg	“Delix 5 1 0 0” OR	Delix 5mg 1-0-0	Delix 5mg 1-0-1
	“Delix 5 1/2 1/2 0”	Delix 5mg 1/2-0-1/2	Delix 5mg 0-0-1/2
		Delix 5-mg 0 1 0	Delix 5 mg 0-1-1/2

### Query token generation

The Anatomical Therapeutic Chemical (ATC) Classification System is an international classification of active ingredients of drugs^6^. In the literature, ATC codes are used to encode drugs and active agents groups. In order to get all brand, drug and agent group names of an ATC-group like *C07 Beta Blocking Agents*, we use the ABDA-DB^7^, which contains all names in English and German. Since medical reports rarely contain the full name of a drug, we processed the names from the ABDA-DB in various ways: a) names were simplified by omitting the names of the manufacturers and the strength of the drug; b) other unnecessary words were removed; that includes modifiers concerning the effect like *forte* and the administration form like *oral*; c) abbreviations and alternative spellings were considered. Table 4 shows examples of the processing of drug names. The resulting tokens were used for the queries. Hyphens do not need to be treated because they are removed by the tokenizing procedure.

**Table 4:** Example for the processing of the drug names

Product name	Processed name	Alternative name
Bayer Aspirin forte 100mg	Aspirin	
Levothyroxin-Natrium	Levothyroxin Natrium	Levothyroxin Na
Paracetamol-Ratiopharm 500mg	Paracetamol	
ACC akut 200mg Hustenlöser	ACC	

### Evaluation

We performed tests to evaluate our development and conducted case studies aiming to replicate findings reported in selected medication trends studies.

#### Medication extraction

Since medication studies only consider the use of drugs, the replication requires just Boolean IE. Therefore we carried out a comprehensive test. We further evaluated the requests for the daily dosage using ad hoc IE. To protect privacy, these texts were de-identified and in addition they must not leave the clinical network.

**Extraction of drugs.** For the evaluation of the medication extraction 600 documents were randomly selected from the disease domains hypertension, atrial fibrillation and chronic kidney disease. From each domain, 100 medication sections from 2005 and 100 sections from 2015 were sampled, resulting in a total of 600 documents. A manually annotated gold standard was created for these documents. All medications, brands, drug and substance names were annotated using the Apache UIMA CAS type system. In order to save time, the text was first automatically pre-announced using the medication tokens gained in “Query token generation” section. Then, the texts were manually corrected to obtain the gold standard. The ATHEN environment^8^ was used to perform this work [28]. Afterwards the original texts were imported into the PaDaWaN-CDW with the data integration pipeline. Then queries were made with all drug names and the hits detected were annotated. At the end, all hits found by the system were compared to the gold standard.

**Daily dosage.** The extraction of the daily medication dosage was evaluated with several drugs: Antihypertensive drugs: EsidrixⓇ (Thiazide-Diuretika, ATC: C03A), ConcorⓇ (*β*-blocker, C07A), DelixⓇ (ACE inhibitor C09A) and novel oral anticoagulants (NOAC) used for atrial fibrillation: EliquisⓇ, PradaxaⓇ, XareltoⓇ. For each drug, 100 medication sections containing this drug from 2015 were selected. For the antihypertensive drugs another 100 units were selected for the year 2005. This was not possible for the NOACs, since they did not exist at that time. Queries were made in the PaDaWaN system and evaluated manually. For the evaluation, all dose strengths were extracted. The proximity query feature was used to extract the dose.

#### Study replication

To evaluate the quality of the study replication, we chose five studies from the literature covering three domains (hypertension, atrial fibrillation, chronic kidney disease) and compared the major and sub-findings with the results of the University Hospital of Würzburg in total, respectively restricted to its Department of Internal Medicine I (Med1) using the ad hoc query feature with of the CDW. The drugs were extracted from the medication section of the discharge letter. That contains in almost every case the medication at discharge representing the recommended / prescribed medication. Additionally the medication at admission is described in 18% (Med1: 13%) of all cases. At discharge from hospital, patients receive 8% (Med1: 19%) more medication than at admission, while nearly all medications from admission were continued at discharge. (Tested for the main drug agent groups for hypertension.) We used the whole medication section with all medication descriptions as data source to identify weather a drug is taken or not.

This was conducted with the PaDaWaN-CDW including about 1 million patients with 5 million patient cases and more than 600 million pieces of single information. We applied the same in- and exclusion criteria as in the respective publications. However, we did not compute age-adjusted values. Not every single evaluation in the publications was reproduced; we rather focused on the main statements and central result tables of the studies or took the most interesting parts of the publications to show the power of our approach.

**Hypertension** We chose [13] as first drug trend study, because it is a highly cited study addressing a large population. The analyzed data was acquired during the National Health and Nutrition Examination Survey (NHANES) [29]. We further aimed to replicate the results of Shah and Stafford [14] concerning the findings on systolic blood pressure. These authors used data from the National Disease an Therapeutic Index (NDTI), a nationally representative physician survey. We extracted this information from the discharge letter via numeric ad hoc IE [18].

**Atrial Fibrillation.** In the replication of the study for atrial fibrillation [3] the ad hoc IE from unstructured texts was combined with structured data from the CDW and differentiated according to these. Subgroups such as comorbidity and age groups were investigated by Gadsbøll et al. [4]. The data sources of these studies were the Danish National Patient Registry, the (Danish) National Prescription Registry and the (Danish) Civil Registration System, containing various information on all prescriptions dispensed in Danish pharmacies since 1995.

**Chronic Kidney Disease.** We also selected a study to examine temporal trends and treatment patterns by patients with CKD and type 2 diabetes mellitus (T2DM) [5]. In this work, medication groups are evaluated. In a more detailed analysis, CKD was broken down into different severity levels (stages), and the medicative effect of the medication groups was considered [5]. This study also used the data from NHANES.

Tables 5 and 6 map all drug and diagnostic group designations used in respective publications to ATC and ICD10 codes, respectively. These codes were used for the replication of these studies. Table 7 summarizes the replicated studies and shows their inclusion and exclusion criteria.

**Table 5:** Mapping between diagnostic group designations used in the literature and ICD10 codes used for the replication

Designation in paper	ICD-10-Code	Abbr.
Abnormal liver function	K77: Liver disorders in diseases classified elsewhere	
Alcohol abuse	F10: Alcohol related disorders	
Atrial fibrillation	I48: Atrial fibrillation and flutter	AF
Bleeding	R58: Hemorrhage, not elsewhere classified	
Chronic kidney disease	N18: Chronic kidney disease	CKD
Deep vein thrombosis	I82: Other venous embolism and thrombosis	
Diabetes mellitus Typ 2	E11: Type 2 diabetes mellitus	T2DM
Heart failure	I50: Heart failure	
Hypertension	I10: Essential (primary) hypertension	HT
Ischemic heart disease	I20-25: Ischemic heart diseases	
Myocardial infarction	I21: Acute myocardial infarction	
Peripheral artery disease	I73.9: Peripheral vascular disease, unspecified	
Pregnant	O00-099: Pregnancy, childbirth and the puerperium	
Pulmonary embolism	I26: Pulmonary embolism	
Stroke	I63: Cerebral infarction	
Valvular disease	I05-I09: Chronic rheumatic heart diseases	
	I34-I37: Nonrheumatic mitral/aortic/tricuspid/pulmonary valve disorders	
	Q22-Q23: Congenital malformations of pulmonary and tricuspid valves / aortic and mitral valves

**Table 6:** Mapping between drug group designations used in the literature and ATC codes used for the replication

Designation in paper	ATC-Codesystem
Insulin	A10A: Insulins and analogues
Oral antidiabetes medication	A10B: Blood glucose lowering drugs, excluding insulins
Biguanides	A10BA: Biguanides
Sulfonylureas	A10BB: Sulfonylureas
Antidiabetes combinations	A10BD: Combinations of oral blood glucose lowering drugs
*α*-Glucosidase inhibitors	A10BF: Alpha glucosidase inhibitors
Thiazolidinediones	A10BG: Thiazolidinediones
DPP-4 inhibitors	A10BH: Dipeptidyl peptidase 4 (DPP-4) inhibitors
Meglitinides	A10BX: Other blood glucose lowering drugs, excluding insulins
Vitamin K antagonists (VKA)	B01AA: Vitamin K antagonists
Warfarin	B01AA03: Warfarin
ADP receptor antagonists	B01AC04: Clopidogrel, B01AC05: Ticlopidine, B01AC22: Prasugrel, B01AC24: Ticagrelor
Oral anticoagulations (OAC)	VKA & NOAC
Non-vitamin K antagonist oral anticoagulants (NOAC)	Dabigatran, Rivaroxaban, and Apixaban
Rivaroxaban	B01AF01: Rivaroxaban
Apixaban	B01AF02: Apixaban
Dabigatran	B01AE07: Dabigatran etexilate
Aspirin	B01AC06 ASS
Dipyridamole	B01AC07: Dipyridamole
Digoxin	C01AA05: Digoxin
Diuretics	C03: Diuretics
Thiazide diuretics	C03A: Low-ceiling diuretics, thiazides
Hydrochlorothiazide	C03AA03: Hydrochlorothiazide
Loop diuretics	C03C: High-ceiling diuretics
Furosemide	C03CA01: Furosemide
Hydrochlorothiazide; triamterene	C03EA01: Hydrochlorothiazide and potassium-sparing agents
*β*-blockers	C07: Beta blocking agents
Metoprolol	C07AB02: Metoprolol
Atenolol	C07AB03: Atenolol
Carvedilol	C07AG02: Carvedilol
Calcium channel blockers	C08: Calcium channel blockers
Amlodipine	C08CA01: Amlodipine
Nifedipine	C08CA05: Nifedipine
Verapamil	C08DA01: Verapamil
Diltiazem	C08DB01: Diltiazem
RAAS	C09: Agents acting on the renin-angiotensin system
Renin-angiotensin system inhibitors:	C09A: ACE inhibitors, plain
Lisinopril	C09AA03: Lisinopril
Lisinopril; hydrochlorothiazide	C09BA03: Lisinopril and diuretics
Angiotensin receptor blockers	C09C: Angiotensin II antagonists, plain
Losartan	C09CA01: Losartan
Valsartan	C09CA03: Valsartan
Olmesartan	C09CA08: Olmesartan medoxomil
Non-steroidal antiinflammatory drugs:	M01A: Anti-inflammatory and antirheumatic products, non-steroids

**Table 7:** Overiew of replicated studies and their inclusion and exclusion criteria

Study topic	Paper	Filters
Hypertension: Trends	[13]	Hypertension, age ≥18, not pregnant
Hypertension: Systolic BP	[14]	Hypertension, 1.1.2014- 1.1.2015
Atrial Fibrillation: Trend & Age Groups	[3]	Atrial Fibrillation, 2005 - 2018, age [30, 100], no valvular disease, no pulmonary embolism, no deep vein thrombosis
Atrial Fibrillation: Characteristics & Brands	[4]	Atrial Fibrillation, 22.8.2011 -1.1.2016, age [30, 100], no valvular disease, no pulmonary embolism, no deep vein thrombosis
CKD & T2DM	[5]	CKD,T2DB, Age ≥18, 2012-2017

## Results

### Ad hoc IE evaluation

#### Extraction of drugs

Table 8 shows the performance of the ad hoc extraction of medications with an overall F1-score of 0.983 (precision 0.997 and recall 0.970).

**Table 8:** Performance of the ad hoc extraction of medications

Dataset	Documents	Medications	TP	FP	FN	Precision	Recall	F1
Overall	600	5701	5529	15	172	0.997	0.970	0.983
2005	300	23000	2176	13	124	0.994	0.946	0.969
2015	300	3041	3353	2	48	0.999	0.986	0.993
I10	200	1817	1768	3	49	0.998	0.973	0.986
I48	200	1795	1741	1	54	0.999	0.970	0.984
N18	200	2089	2020	11	69	0.995	0.967	0.981

Most errors were caused by abbreviations. The misspelling based errors could be significantly reduced by the error tolerant query feature. Table 9 shows the error analysis of the ad hoc extraction of medications. The most common occurrences of the error groups are shown below. Abbreviation Fraxi (20), Tiotropium (6), Mg Verla (4), Dreisavit (3), Dabigatran (2), Insuman (2), Isosorbid (2) Not in DB Eunerpan (9), Polybion (4), Aclidinium (2), Calcetat (2), Natriumperchlorat (2), Cranoc (2), Calcetat (2) Alternative notation Glycopyrronium (2), Dikalium Clorazepat (2), Humaninsulin (1), Diuretikum (1), Ca Carbonat (1) Misspelling Ferrosanol (4), Eins alpha (2), Amphomoronal (2), Beclometasondipropionat (2), Klazid (2), Rehnagel (2), Cardular (2), Calciumdiacetat (2) Search to fuzzy diabetes ≈ diabetex (4), diagnostik ≈ diagnostika (1), antihypertensiven ≈ antihypertensives (1) Incorrect extracted medication thrombozyten (1), cholesterin (1), albumin (1), kalium (1), natrium (1)

**Table 9:** Error analysis of the ad hoc extraction of medications

	Medications		Occurrences	
	#	%	#	%
Abbreviation	40	33%	76	41%
Not in DB	22	18%	39	21%
Alternative notation	9	7%	10	5%
Misspelling	38	31%	47	25%
Search to fuzzy	3	2%	6	3%
Incorrect extracted medication	9	7%	9	5%

#### Extraction of daily drug dose

An analysis on the data set for the daily dose, that contains 900 mentions of selected drugs, revealed that 5% of the mentioned drugs were discontinued or reduced. 90% had an indicated strength, 92% an instruction and 89% a strength and an instruction. See Table 10.

**Table 10:** Presence of strength and instruction application of medication in the evaluation set

	#	%
Intake (not discontinued)	852	95%
With strength	814	90%
With instruction	829	92%
With strength and instruction	800	89%

The most common daily taken dose was one unit (57%) followed by two units (31%), see Table 11.

**Table 11:** Summed daily dose of the medication units in the evaluation set

Daily units	#	%
0.25	1	0.1%
0.5	85	10.0%
1	489	57.4%
1.5	7	0.8%
2	264	31.0%
3	5	0.6%
4	1	0.1%

The overall F1-score for the extraction of the daily medication dose was 0.974. The precision was the same or slightly higher than the recall in all tests. The extraction results were slightly better on the antihypertensive drug set (F1: 0.982) than on the NOACs drug set (F1: 0.958). The documents from 2015 also showed slightly better results than those of 2005 (F1: 0.977 vs 0.968). The complete results can be found in Table 12.

**Table 12:** Performance of the ad hoc extraction of the daily medications dose

Dataset	Documents	TP	FP	FN	Precision	Recall	F1
Overall	900	875	21	25	0.977	0.972	0.974
Xarelto	100	100	0	0	1.0	1.0	1.0
Eliquis	100	95	3	5	0.960	0.950	0.955
Pradaxa	100	92	6	8	0.939	0.920	0.929
NOACs	300	287	12	13	0.960	0.957	0.958
Esidrix	200	197	2	3	0.990	0.985	0.987
Concor	200	196	4	4	0.980	0.980	0.980
Delix	200	195	3	5	0.985	0.975	0.980
Antihypertensive drug	600	581	9	12	0.985	0.980	0.982
2015	600	586	13	14	0.978	0.977	0.977
2005	300	289	8	11	0.973	0.963	0.968

Most errors were caused by an unusual notation. See Table 13 and listing below. Other error sources were supplements, which contained numbers, incorrect splitting of the tokenizer, double mentions in same document, segmentation faults, and a too wide gap between the drug name and the instructions. Notation Esidrix 1x1, Pradaxa 150-0-150 mg Supplement Pradaxa 110 mg 1-0-1 (bitte 1 Tag vor stationären Aufnahmetermin pausieren); Tokenizer EuthyroxⓇDouble mention Medikation bei Entlassung: Esidrix 12,5 mg 1-0-0; Medikamente bei Entlassung: Esidrix 25 pausiert Segmentation Gap Concor 5 mg (bei Bedarf) 1 – 0 – 0 – 1

**Table 13:** Error analysis of the ad hoc extraction of the daily medications dose

Error	#	%
Notation	23	50%
Supplement	6	13%
Tokenizer	6	13%
Doublet	5	11%
Segmentation	4	9%
GAP	2	4%

### Study replication

The presented results for the University Hospital of Würzburg (UKW) and the Department of Internal Medicine I (Med1) were computed via ad hoc IE (see “Study replication” section). Since the ad hoc IE had an F1 score of 0.974, there may be small deviations from the exact values.

#### Hypertension

**Study: Trends in antihypertensive medication use and blood pressure control among United States adults with hypertension** Table 14 shows the results of the replication of the medication trend study to hypertension for the years 2000 to 2010. The findings of the referenced paper and their reproducibility by our results are listed in Table 15. The computation time to query the data for Table 14 from the CDW was 2 min 26 s.

**Table 14:** Replication of the medication group trend study for hypertension [13]

		2000 -2001	2003 -2004	2005 -2006	2007 -2008	2009 -2010	Overall
n	Paper	1669	1750	1564	2169	2168	9320
	UKW	4720	12267	17823	20187	23646	78643
	Med1	3485	5938	6690	7596	9189	32898
Diuretics	Paper	30%	32%	34%	35%	36%	34%
	UKW	48%	46%	45%	46%	48%	46%
	Med1	48%	56%	61%	60%	59%	58%
Thiazide-Diuretics	Paper	22%	24%	26%	27%	28%	26%
	UKW	14%	21%	20%	18%	18%	18%
	Med1	13%	24%	24%	20%	17%	20%
*β*-blockers	Paper	20%	25%	30%	28%	32%	27%
	UKW	58%	52%	50%	52%	56%	53%
	Med1	62%	69%	73%	72%	71%	70%
CC-Blocker	Paper	19%	21%	22%	19%	21%	20%
	UKW	27%	24%	24%	25%	28%	26%
	Med1	27%	30%	33%	34%	36%	33%
ACE inhibitors	Paper	26%	30%	29%	29%	33%	30%
	UKW	49%	46%	42%	44%	46%	45%
	Med1	51%	57%	56%	57%	55%	56%
ARB	Paper	11%	15%	15%	20%	22%	17%
	UKW	10%	11%	13%	14%	16%	14%
	Med1	11%	14%	16%	19%	20%	17%

Drug agent groups compared to the reference paper with all patients and Med1 clinic patients from University Hospital of Würzburg (UKW) during 2000-2010

**Table 15:** Findings of the replicated studies compared to our results

	Finding	Rep.
	**Main findings**	
1	Any antihypertensive drug increased	(Yes)
	**Other findings**	
2	diuretics remained the most commonly used antihypertensive drug class	No
3	more than one third of hypertensive adults reported taking diuretics	Yes
4	Use of thiazide diuretics accounted for three fourths of all diuretic use.	No
5	The prevalence of thiazide diuretic use increased slightly	Yes
6	The overall prevalence of use of *β*-blockers increased	Yes
7	Approximately 20% use CCBs in each survey period	Yes
8	the use of CCBs remained relatively constant	Yes
9	ACE inhibitors were the second most commonly used antihypertensive drug class	No
10	The use of ACE inhibitors increased significantly overall.	No
11	The use of ARB increased significantly	Yes

Study: Trends in antihypertensive medication use and blood pressure control among United States adults with hypertension clinical perspective

**Current trends of hypertension treatment in the United States.**

Table 16 shows the grouped systolic blood pressure of hypertensive patients and Table 18 lists their the use of drug agent groups. The findings of the referenced paper and their reproducibility by our results are listed in Table 17. The computation time to query the data for Tables 16 and 18 from the CDW was aggregated 49 min 55 s.

**Table 16:** Systolic blood pressure (SBP) in mm Hg of hypertensive patients compared to [14]

	<130	[130−139]	[140−149]	[150−159]	≥160
Paper	32%	26%	19%	9%	15%
UKW	23%	12%	11%	10%	45%
Med1	25%	13%	11%	9%	42%

**Table 17:** Findings of the replicated studies compared to our results

	Finding	Rep.
	**Main finding**	
1	BP control widely varied among this medication-treated group of patients.	Yes
	**Other findings**	
2	ACEI use was significantly more likely in patients with SBP <130 compared with those with BP ≥160.	No
3	The use of CCBs was less likely among those with SBP <130, but more likely among those with SBP ≥160	Yes

Study: Current trends of hypertension treatment in the United States

**Table 18:** Use of drug agent groups and systolic blood pressure (SBP, measured in mm Hg) groups of hypertensive patients compared to [14]

SBP		Thiazide	*β*-Blocker	CCB	ACEI	ARB
<130	Paper	25,1%	20,4%	20,0%	31,1%	21,1%
	UKW	14,3%	61,7%	27,3%	38,6%	21,4%
	Med1	15,5%	67,0%	30,8%	38,0%	23,1%
[130-139]	Paper	27,8%	17,2%	23,1%	29,7%	22,3%
	UKW	14,9%	54,7%	35,4%	42,9%	24,2%
	Med1	13,3%	61,9%	40,7%	44,2%	27,4%
[140-149]	Paper	24,7%	17,8%	23,7%	27,7%	22,5%
	UKW	17,2%	52,4%	33,1%	44,1%	24,8%
	Med1	17,0%	67,0%	41,5%	45,7%	34,0%
[150-159]	Paper	25,4%	17,9%	24,9%	25,6%	23,0%
	UKW	22,9%	52,7%	38,9%	48,9%	23,7%
	Med1	22,9%	61,4%	48,2%	54,2%	21,7%
≥160	Paper	26,0%	20,6%	26,0%	25,4%	20,5%
	UKW	22,9%	51,4%	37,0%	52,1%	23,4%
	Med1	16,5%	57,4%	41,2%	51,6%	23,9%

#### Chronic kidney disease

**Fig. 2:**
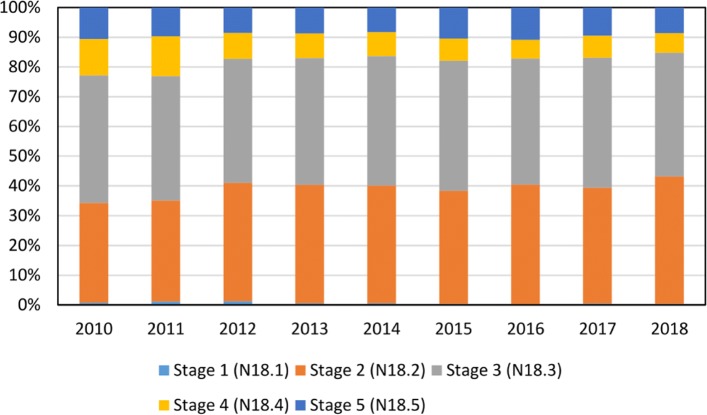
Temporal trend of CKD stages in the UKW. The severity degrees of CKD-patients are shown over time

**Study: Understanding CKD among patients with T2DM: prevalence, temporal trends, and treatment patterns – NHANES 2007-2012** Figure 2 is an additional evaluation showing all severity levels of CKD over time. The computation time to query the data from the CDW was 14 s.

**Fig. 3:**
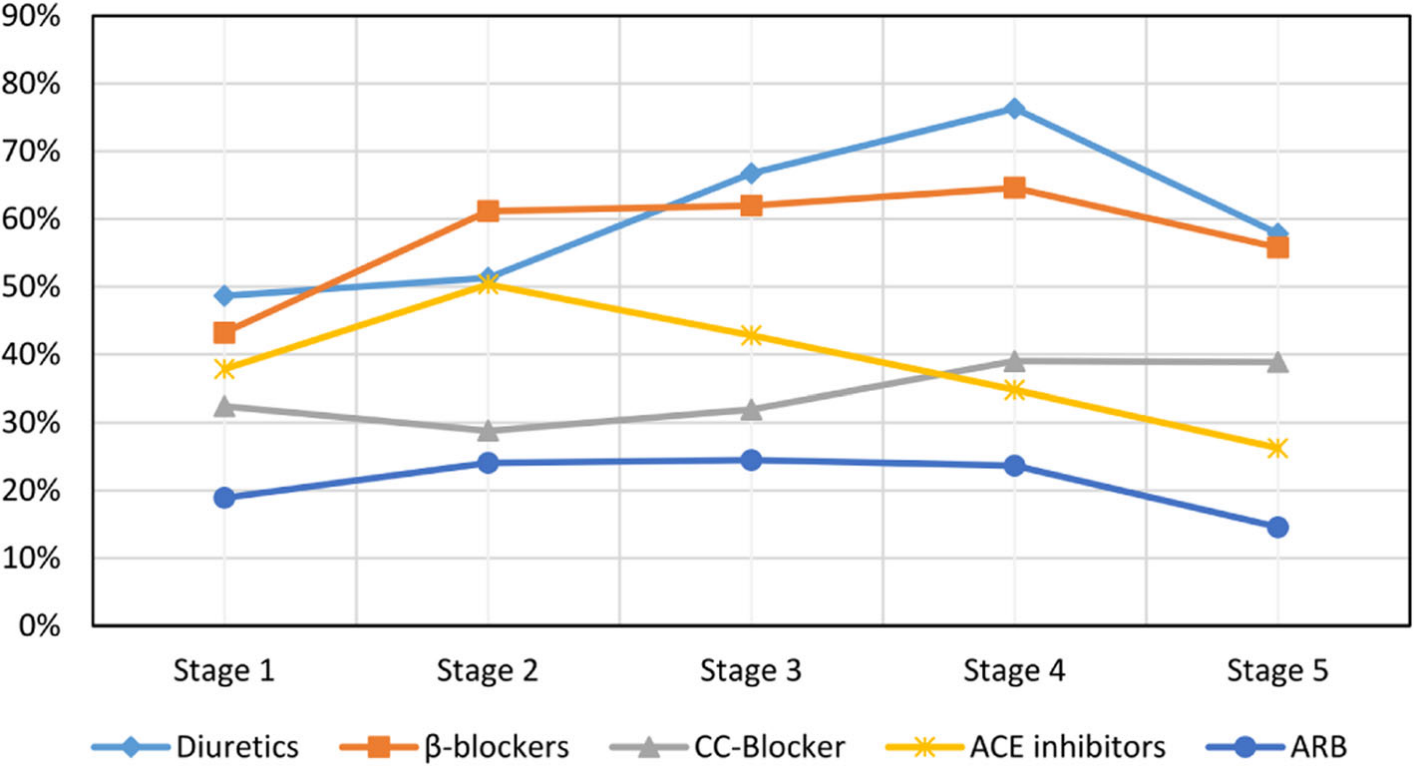
Medication agent groups by degrees of severity of CKD in the UKW of CKD patients with hypertension

Figure 3 shows the hypertension medication agent groups by degrees of severity of CKD for all patients with hypertension and CKD for the years 2013-2016. The computation time to query the data from the CDW for Fig. 3 was 1 min 3 s.

Tables 19 and 21 compare the findings of Wu et al. [5] to our findings for the UKW and the Med1 concerning medication and agent groups for patients with CKD and T2DM. It shows the medication for diabetes as well as the hypertension. The findings of the referenced paper and their reproducibility by our results are listed in Table 20. The computation time to query the data from the CDW was 3 min 16 s for Table 19 and 5 min 9 s for Table 21.

**Table 19:** Medication and agent groups for CKD with T2DM compared to [5]

	Overall	No CKD	Stage 1	Stage 2	Stage 3	Stage 4	Stage 5
n							
Paper	1380	1122	144	159	258	32	16
UKW	35636	20314	34	4725	7659	1671	1603
Med1	13461	6452	*	2264	3319	735	766
DM medication							
Paper	83%	81%	84%	89%	84%	94%	77%
UKW	60%	59%	59%	69%	62%	55%	44%
Med1	71%	69%	*	79%	72%	69%	61%
Insulin							
Paper	19%	15%	16%	28%	24%	38%	63%
UKW	26%	24%	24%	23%	30%	38%	35%
Med1	38%	39%	*	28%	39%	52%	51%
Oral antidiabetes medication							
Paper	75%	75%	81%	77%	72%	69%	44%
UKW	46%	47%	41%	59%	46%	28%	13%
Med1	51%	50%	*	69%	52%	31%	16%
Biguanides							
Paper	56%	62%	68%	55%	36%	4%	3%
UKW	32%	34%	26%	48%	27%	7%	1%
Med1	34%	33%	*	57%	32%	6%	0%
Sulfonylureas							
Paper	35%	31%	44%	42%	42%	56%	15%
UKW	8%	7%	9%	10%	10%	7%	2%
Med1	7%	6%	*	11%	9%	7%	2%
DPP-4 inhibitors							
Paper	7%	7%	4%	8%	8%	23%	7%
UKW	12%	11%	24%	14%	17%	13%	7%
Med1	17%	15%	*	19%	20%	17%	10%

Values with * were omitted due to small sample sizes

**Table 20:** Findings of the replicated studies compared to our results

	Finding	Rep.
	**Main findings**: The use of antidiabetic and antihypertensive medications generally followed treatment guideline recommendations:	
1	The use of metformin was significantly limited with increasing CKD severity	Yes
2	The use of insulin increased sharply in severe CKD stages	Yes
3	Antihypertensive medications were used extensively	Yes
4	The level of RAAS inhibitor (including ACE inhibitors and ARBs) use was consistent, even in patients without CKD and with mild-to-moderate CKD	Yes
5	Use of thiazide diuretics was more prevalent than other diuretic agents with mild-to-moderate CKD	Yes
6	Thiazide diuretics were replaced by loop diuretics among those with moderate CKD to kidney failure	Yes
	**Other findings**	
	*Antidiabetes medications:*	
7	Overall, 83.1% of individuals with T2DM received antidiabetic medications	No
8	The use of insulin, biguanide (metformin), and sulfonylurea (SU) was significantly different between patients without CKD, those with mild-to-moderate CKD, and those with moderate CKD to kidney failure	Yes
9	The use of dipeptidyl peptidase-4 (DPP-4) inhibitors was similar	Yes
10	The use of sulfonylurea (SU)s increased in later CKD stages (3b and 4)	No
11	Sulfonylurea SU use dropped in CKD stage 5	Yes
	*Antihypertensive medications:*	
12	Overall, 75.7% of individuals with T2DM received antihypertensive medications	Yes
13	Use was extensive in those with CKD stage 2 or higher	Yes
14	Fewer than two-thirds were taking some form of RAAS inhibitor	(Yes)
15	There was a difference in the use of ACE inhibitors and ARBs between patients without CKD, those with mild-to-moderate CKD, and those with moderate CKD to kidney failure	Yes
16	The use of *β*-blockers, diuretics, and CCBs was statistically different	Yes
17	ARBs appeared to be more commonly used in stages 3a–4	Yes
18	The use of *β*-blocker and CCBs trended upward with increasing CKD severity	(Yes)
19	Diuretic use also increased from stage 1 through stage 4, but sharply fell in stage 5	Yes
20	Dhiazide diuretics were more commonly used by individuals without CKD or with mild-to-moderate CKD compared with other diuretic subclasses	Yes
21	In later CKD stages, the dominance of thiazide diuretics was replaced with loop diuretics	Yes
22	*β*-Blocker use increased with stages 4 and 5 CKD	No

Study: Understanding CKD among patients with T2DM: prevalence, temporal trends, and treatment patterns—NHANES 2007–2012

**Table 21:** Medication and agent groups for CKD with T2DM compared to [5]

	Overall	No N18	Stage 1	Stage 2	Stage 3	Stage 4	Stage 5
n							
Paper	1380	1122	144	159	258	32	16
UKW	10314	15315	34	4723	7656	1671	1601
Med1	6452	7009	*	2266	3319	734	765
Hypertension medication							
Paper	76%	69%	63%	90%	92%	100%	97%
UKW	77%	68%	71%	89%	90%	89%	79%
Med1	85%	75%	*	96%	96%	96%	90%
Diuretics							
Paper	36%	30%	22%	42%	58%	76%	34%
UKW	53%	39%	56%	60%	76%	82%	64%
Med1	63%	47%	*	65%	84%	90%	76%
Thiazide diuretics							
Paper	24%	23%	18%	24%	30%	33%	0%
UKW	14%	13%	24%	22%	15%	10%	2%
Med1	12%	10%	*	23%	14%	7%	1%
Loop diuretics							
Paper	14%	7%	3%	21%	31%	54%	34%
UKW	40%	26%	41%	40%	64%	78%	63%
Med1	51%	36%	*	43%	74%	88%	74%
Potassium-sparing diuretics							
Paper	6%	6%	1%	4%	7%	8%	9%
UKW	11%	8%	6%	14%	20%	14%	6%
Med1	16%	11%	*	18%	27%	16%	9%
*β*-blockers							
Paper	31%	24%	15%	45%	46%	76%	82%
UKW	52%	43%	38%	62%	66%	68%	58%
Med1	64%	52%	*	74%	77%	78%	71%
CC-Blocker							
Paper	20%	15%	13%	37%	25%	33%	57%
UKW	29%	24%	29%	33%	35%	43%	37%
Med1	34%	28%	*	36%	39%	50%	45%
ACE inhibitors							
Paper	40%	38%	43%	51%	42%	28%	41%
UKW	38%	35%	41%	50%	44%	34%	27%
Med1	43%	38%	*	56%	48%	37%	32%
ARB							
Paper	22%	19%	11%	25%	32%	35%	16%
UKW	19%	16%	18%	24%	26%	25%	15%
Med1	24%	19%	*	30%	32%	32%	18%
RAAS							
UKW	58%	52%	59%	74%	69%	59%	42%
Med1	68%	58%	*	86%	80%	68%	50%

#### Atrial fibrillation

The studies on atrial fibrillation (AF) investigate the characteristics and the temporal trend of the use of oral anticoagulants (OAC).

**Fig. 4:**
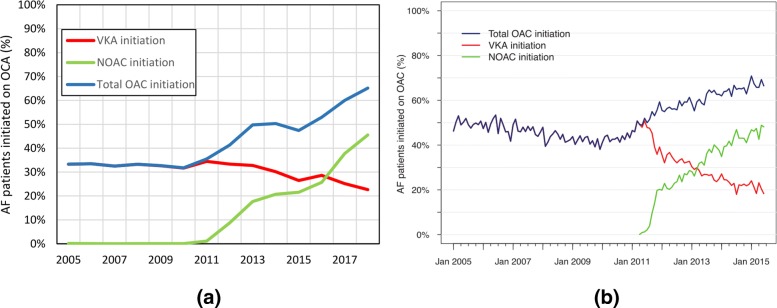
Temporal trend of VKA and OACs compared to [4]. **a** UKW. **b** Paper

**Study: Increased use of oral anticoagulants in patients with atrial fibrillation: temporal trends from 2005 to 2015 in Denmark**Gadsbøll et al. investigate the increased use of oral anticoagulants in patients with atrial fibrillation [3]. Figure 4 shows the temporal trend of VKA and OACs compared to [4]. The findings of the referenced paper and their reproducibility by our results are listed in Table 22. The computation time to query the data from the CDW for Fig. 4 was 25 s.

**Table 22:** Findings of the replicated studies compared to our results

	Finding	Rep.
	**Main findings**	
1	since 2010, more incident AF patients were initiated on OAC treatment	Yes
2	NOACs have replaced VKA as the OAC of choice in AF	Yes
	**Other results**	
3	OAC initiation rates among the incident AF patients decreased from January 2005 to December 2009	Yes
4	From 2010, more patients were initiated on OAC therapy	Yes
5	From 2011, more prevalent AF patients were treated with an OAC	Yes
6	From 2011, a decreasing proportion of the newly diagnosed AF patients was initiated on VKA	Yes
7	This decrease in VKA initiation was followed by a rapid increase in NOAC initiation	Yes

Study: Increased use of oral anticoagulants in patients with atrial fibrillation: temporal trends from 2005 to 2015 in Denmark

**Fig. 5:**
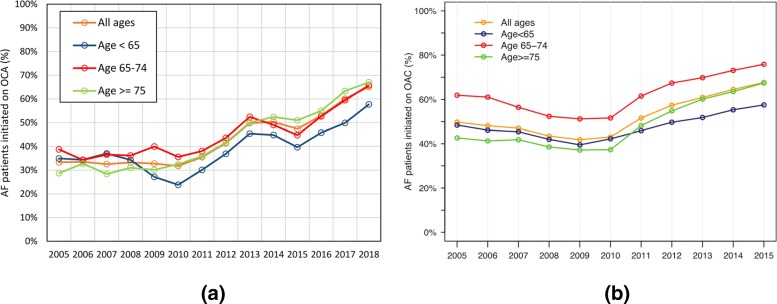
Temporal trend of OAC clustered by age groups compared to [4]. **a** UKW. **b** Paper

Figure 5 shows the temporal trend for AF patient age groups using OACs like in [4]. The computation time to query the data from the CDW for Fig. 5 was 55 s.

**Fig. 6:**
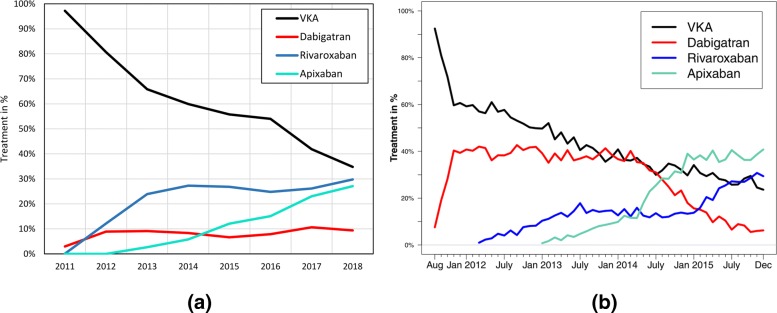
Temporal trend of VKA and OAC usage of all AF patients compared to [4]. **a** UKW. **b** Paper

**Fig. 7:**
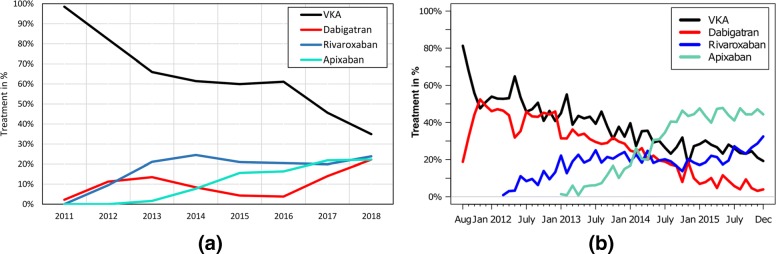
Temporal trend of VKA and NOACs of AF patients aged ≥85 compared to [4]. **a** UKW. **b** Paper

**Study: Non-vitamin K antagonist oral anticoagulation usage according to age among patients with atrial fibrillation: Temporal trends 2011–2015 in Denmark** Staerk et al. made a detailed research for the years 2011 and 2015, since NOAC became relevant [4]. Figures 6 and 7 is a detailed analyses of the temporal trend OACs listing its representatives: Dabigatran, Rivaroxaban, Apixaban. The computation time to query the data from the CDW was 36 sec for Fig. 6 and 29 sec for Fig. 7.

Table 24 shows the distribution among sex and age groups. Table 25 analyses the comorbidities and Table 26 lists the concomitant medication. The values in the referenced paper refer to the time period between 22.8.2011 and 1.1.2016. We computed the values for the same period (named UKW_11) and for the period 1.1.2016 - 1.1.2018 (named UKW_16). The computation time to query the data from the CDW was 1 min 10 s for Table 24, 1 min 40 s for Table 25 and 2 min 10 s for Table 26. The findings of the referenced paper and their reproducibility by our results are listed in Table 23.

**Table 23:** Findings of the replicated studies compared to our results

	Finding	Rep.
	**Main findings**	
1	The absolute number of patients initiating OAC has increased among patients aged <65, 65 to 74, and ≥85 years	yes
2	The utilization of VKAs has decreased since the introduction of NOACs	yes
3	From 2014 [to 2015] the utilization of dabigatran has decreased, especially among patients aged ≥85 years	yes
4	Apixaban has increased significantly and was the most used NOAC drug among patients aged ≥85 years	(yes)
	**Other results**	
5	For patients aged 75 to 84 years, number of patients initiating OAC treatment stayed approximately the same	no
6	The utilization of dabigatran increased within a couple of months since its introduction to the market	yes
7	A fairly constant level of dabigatran utilization was seen from December 2011 of approximately 40%	no
8	Rivaroxaban has steadily increased usage and at study end 29%	yes

Study: Non-vitamin K antagonist oral anticoagulation usage according to age among patients with atrial fibrillation: Temporal trends 2011–2015 in Denmark

**Table 24:** Characteristics of patients with atrial fibrillation using VKAs or OAC medications compared to [4]

		VKA	Dabigatran	Rivaroxaban	Apixaban
N (%)	Paper	42%	29%	13%	16%
	UKW_11	66%	8%	22%	6%
	UKW_16	48%	9%	26%	19%
Males (%)	Paper	57%	55%	50%	50%
	UKW_11	59%	62%	61%	63%
	UKW_16	61%	66%	62%	58%
Age <65	Paper	22%	24%	17%	15%
	UKW_11	12%	21%	25%	17%
	UKW_16	10%	9%	21%	15%
Age 65 to 74	Paper	33%	35%	33%	31%
	UKW_11	28%	29%	28%	22%
	UKW_16	25%	25%	29%	25%
Age 75 to 84	Paper	31%	28%	29%	31%
	UKW_11	45%	35%	34%	40%
	UKW_16	46%	49%	36%	42%
Age ≥85	Paper	13%	13%	21%	22%
	UKW_11	15%	15%	13%	21%
	UKW_16	19%	17%	14%	18%

**Table 25:** Comorbidities of patients with atrial fibrillation using VKAs or OAC. (Continuation of Table 24)

		VKA	Dabigatran	Rivaroxaban	Apixaban
Stroke	Paper	15%	15%	18%	21%
	UKW_11	2%	13%	5%	13%
	UKW_16	3%	26%	3%	2%
Myocardial infarction	Paper	11%	7%	6%	7%
	UKW_11	3%	1%	2%	1%
	UKW_16	2%	2%	4%	1%
Ischemic heart disease	Paper	26%	20%	20%	21%
	UKW_11	32%	26%	23%	31%
	UKW_16	29%	29%	31%	30%
Heart failure	Paper	19%	14%	15%	16%
	UKW_11	31%	25%	26%	34%
	UKW_16	35%	26%	31%	38%
Diabetes mellitus	Paper	14%	11%	12%	13%
	UKW_11	32%	22%	22%	28%
	UKW_16	32%	24%	23%	29%
Hypertension	Paper	47%	44%	44%	43%
	UKW_11	69%	68%	63%	67%
	UKW_16	67%	71%	61%	64%
Chronic kidney disease	Paper	8%	2%	4%	5%
	UKW_11	58%	54%	49%	51%
	UKW_16	49%	43%	46%	49%

**Table 26:** Concomitant medication of patients with atrial fibrillation using VKAs or OAC. (Continuation of Table 24)

		VKA	Dabigatran	Rivaroxaban	Apixaban
ADP receptor antagonists	Paper	10%	8%	10%	11%
	UKW_11	4%	8%	3%	4%
	UKW_16	5%	10%	11%	3%
ASS	Paper	43%	38%	38%	36%
	UKW_11	11%	15%	13%	11%
	UKW_16	9%	15%	11%	8%
Non-steroidal antiinflammatory drugs	Paper	15%	15%	14%	14%
	UKW_11	6%	5%	5%	3%
	UKW_16	8%	9%	8%	5%
Loop diuretics	Paper	22%	15%	18%	19%
	UKW_11	59%	42%	42%	52%
	UKW_16	60%	40%	41%	54%
Beta-blockers	Paper	45%	38%	39%	37%
	UKW_11	77%	76%	77%	78%
	UKW_16	77%	72%	75%	76%
Calcium channel blockers	Paper	29%	26%	27%	26%
	UKW_11	32%	29%	30%	30%
	UKW_16	32%	33%	29%	28%
Renin-angiotensin system inhibitors	Paper	43%	42%	41%	43%
	UKW_11	46%	40%	38%	42%
	UKW_16	39%	42%	35%	38%

Table 27 summarizes the results of the study replication. Main findings were replicated and confirmed by us to 93%, sub-findings to 68% and overall to 75%.

**Table 27:** Summary of the of the study replication results, including main, sub and overall findings

Paper topic	Ref	Main finding	Sub finding	Overall
HT: Trends	[13]	50%	50%	50%
HT: SBP	[14]	100%	50%	67%
CKD & T2DM	[5]	75%	75%	82%
AF Trend 2005-2015	[3]	100%	100%	100%
AF: Characteristics & Brands	[4]	88%	50%	69%
**Overall**		**93%**	**68%**	**75%**

The table shows the amount of findings, which were replicated and confirmed by us

**Daily medication dose extraction.**

As an additional evaluation, we extracted the daily dose of patients with AF using ad hoc IE. All three OACs agent groups with their drugs where analyzed: Xarelto (Rivaroxaban) (see Table 28), Eliquis (Apixaban) (see Table 29) and Pradaxa (Dabigatran) (see Table 30).

**Table 28:** Extraction of the daily medication dose of Xarelto for patients with AF

d. u.	10 mg	15 mg	20 mg	50 mg
1	0,9%	26,6%	67,4%	0,5%
1,5	0,0%	0,0%	0,0%	0,0%
2	1,4%	1,4%	1,4%	0,0%
3	0,0%	0,0%	0,5%	0,0%
Sum	2,3%	28,0%	69,3%	0,5%

Average dose: 19,3 mg

**Table 29:** Extraction of the daily medication dose of Eliquis for patients with AF

d. u.	2,5 mg	5 mg
1	3,7%	3,2%
1,5	0,0%	0,0%
2	43,2%	49,5%
3	0,0%	0,5%
Sum	46,8%	53,2%

Average dose: 7,4 mg

**Table 30:** Extraction of the daily medication dose of Pradaxa for patients with AF

Daily units	10 mg	75 mg	110 mg	150 mg
1	0,0%	1,1%	5,6%	3,3%
1,5	0,0%	0,0%	0,0%	0,0%
2	1,1%	3,9%	51,1%	33,3%
3	0,0%	0,0%	0,6%	0,0%
Sum	1,1%	5,0%	57,2%	36,7%

Average dose: 232,3 mg

The average daily dose was 19,31 mg of Xarelto, 7,4 mg of Eliquis and 232,3 mg of Pradaxa.

## Discussion

First, the results of the replication studies are discussed, and second, the ad hoc IE tests and the system itself are compared to other approaches.

### Study replication

**Major result & comparison.** One study (AF Trend from 2005 to 2015 [3]) could be completely replicated, i.e., all main findings and sub-findings were confirmed by us. Overall, 93% of the main findings, 68% of other detailed findings and 75% of all findings could be replicated. Table 27 lists the results of the individual replications. As mentioned in “Background” section, many researchers have tried to reproduce other researchers work, but 70% failed. 24% researchers reporting a successful replication of experiments were able to publish their work. In case of unsuccessful reproduction this proportion was only 13% [16]. Of course, when conducting replication experiments, some deviations have to be expected. Concerning the sources of variation, not only the exact reproduction of the study design is important, but also the population under study and time trends observed regarding diagnosis and therapy matter. E.g., Gu et al. reported that the control of blood pressure (BP) levels “varied greatly between recent publications” [13]. Staerk et al. mentioned that the most frequently used NOAC agent in their study was different to a previous study owing to changes in prescription patterns over time [4].

**Study details.** The distribution among the groups of active substances for hypertension in the UKW was slightly different compared to the paper [13]. In Med1, patients got substantially more drugs, probably indicating treatment preferences of a certain clinic.

In the CKD study, 75% of all findings agreed with our results, but there were also some deviations. Some observations differed only in stage 5 of CKD. This could be explained with different sizes of population of the subgroups with level 1, 4 and 5. These were caused by the basic population (population-based sample vs. hospital patients). The trends in the studies of atrial fibrillation could be replicated by us, however with a surprisingly small temporal shift. The comorbidities and the concomitant medication differed slightly, but many agreed.

**Data acquisition & study population.** The studies differed regarding the data acquisition approach: The hypertension [13] and CKD [5] studies were based on NHANES, the AF studies [3, 4] on the Danish National Prescription Registry and the hypertensive study with SBP used a physician survey. The medication in NHANES was "self-reported data (via a patient survey questionnaire)" [5]. We took the medication information from the discharge letter written by physician, which should be reflected in higher accuracy. NHANES is a representative sample of the U.S., i.e. both healthy and sick people, whereas a CDW collects information on hospitalized or ambulatory patients. There are even differences within a hospital. The medication use was found higher in almost all cases at the Med1 compared to the entire clinic. This is comprehensible, because hypertension, atrial fibrillation and chronic kidney diseases are usually treated there. The studies also differed regarding the number of analyzed cases. The AF studies used a nation-wide data source, i.e. three to four times more patients than which were present in the local CDW. For the hypertension study, we analyzed eight times more cases, in the CKD even 25 times more cases.

**Analysis duration.** While our queries took only a few minutes, it probably took a few weeks or months to conduct the studies for the referenced papers.

### Ad hoc IE

Ad hoc IE possesses features of a conventional IE and query functions of CDWs. Therefore, the evaluation results and the system itself are compared with other approaches.

#### Comparison of evaluation results

According to [22] MedEx is the most widespread used tool for extracting medication information from clinical texts. In their original paper they achieved an F1-score of 93,2% for extracting drug names, a score of 94,6% for the strength and 96,0% for the frequency [19]. Two years later they published a case study around the medication *warfarin* and pushed the F1 score to 95% (recall 99,7%, precision 90,8%) for extracting the daily dosage [30]. In another study, they tried to calculate the daily dosage for the drug *tacrolimus* with an extended MedEx version and reported precisions of 90-100% and recalls of 81-100%. For discharge summaries they achieved F1 measures of 96% for strength and 88% for daily dosage [31].

Some papers mention, that they had to deal with more complex medication instructions like dosing in 2 h intervals [19, 30–32]. This may complicate the calculation of the dosage and explain the inferior results compared to ours (F1 97,4%, precision 97,7%, recall 97,2%).

The results of the extraction of the drug names alone were only partially comparable with ours. First, no lists of medications were used in the literature, and second, these are all conventional IEs. We applied ad hoc IE, which extracts the information on the fly during runtime.

### Conventional versus ad hoc IE

**Conventional IE.** IE turns unstructured information embedded in texts into structured data [33]. More precisely, it is the automatic extraction of concepts, entities and events, as well as their relations and associated attributes [22]. It consists of subtasks, i.e. entity recognition, relation extraction, event extraction (including time and date), and template filling [33]. In a conventional IE application information are computed by many expensive processing steps [34]. Therefore, each text is annotated several times, e.g. with parts of speech tagging, syntactic or dependency parsing or word list labeling. The output of a tagging process is the input for the next step. Thereafter rule-based systems apply rules on these annotations to extract information. Machine learning approaches use additional features and a trained model for the extraction step.

**Ad hoc IE.** In ad hoc IE, a segmentation separates non-related concepts. On these segments, a one-step annotation can be made effectively. But this step is quite fast, due to the index, and in contrast to the conventional IE, there are not “many of expensive processing steps” [34]. Thus, ad hoc IE is suitable for domains that can be handled with a one-step annotation. A survey revealed that 65% of clinical information extraction systems are rule-based and often use a regular expression as a search pattern [22]. Hence, they are interesting for ad hoc IE and could possibly be implemented with it. Ad hoc IE shifts the time of extraction from the data-integration phase to runtime, enabling a flexible IE at runtime for all users.

Ad hoc IE does not address all sub-tasks of a conventional IE application. However, the tasks important to the medical domain are supported: Named entity recognition is ensured by the query functions, relation extraction for medical concepts is accomplished by segmentation and for patient identification by context detection.

**Comparison** In summary, the ad hoc IE was found to be very well suited for this task. It yielded as good results as the conventional IE but was characterized by a much lower developmental effort, promptness of results and intuitive adaptability by users. In domains with complicated structure, conventional IE might be superior in terms of confidence and accuracy [18]. However, ad hoc IE does not claim to replace conventional IE, it rather should be considered a supplement for quick analysis to get a good and detailed overview for further investigations. An additional advantage of ad hoc IE is its ability not only to return the number of hits, but also to retrieve hit snippets from texts. This addresses two points: 1) Queries can be refined iteratively and 2) the system can also be used as an evaluation environment.

#### Query Features of other CDWs

Text query features are poorly supported in CDWs [18]. Most of them, like the well known i2b2, store their data in SQL-DBs and just support the *like*-operator^9^ a SQL full text index. Other CDW index their textual data with index libraries as Apache Solr (e.g. tranSMART [35] or Roogle [36]) or with SQL full text index (e.g. STRIDE [37]). Dr. Warehouse performs an negation detection as well and excludes negated findings from the search [38]. However, no system has query features that exceed a token search.

**Comparison to SQL** Many CDWs use a SQL-Server as storage engine. Texts can be queried via the *like*-operator, which is used to perform wildcard queries. However, this is limited in many ways: Error tolerant queries, which deal with misspellings, are not supported. Drug names that consist of several words are difficult or cumbersome to find with SQL methods. Especially, if these words are not next to each other and, e.g., separated by a brand name.

Extracting dose information reliably using SQL is next to impossible. Several words can be between the drug name and the instruction, e.g. additional information about the application. A segmentation of the drugs would be necessary in any case. Additionally, an SQL-based approach is much slower than a text index based system.

### Limitations

Limitations for conducting medication trend studies in a CDW relate to complex inclusion and exclusion criteria that can not appropriately be mapped, like complex temporal constraints. Some techniques frequently used in clinical analyses are more difficult to apply like adjustment for important confounders, e.g. sex and age. This is not a technical limitation, but it would require a laborious recalculation.

The feasibility of replication studies depends as well on the data embedded in the CDW. Only integrated concepts or texts can be queried. The populations of studies are always different, so the population of a specific hospital department does not correspond to the overall population.

## Conclusion

With the presented approach of the ad hoc IE for medications, which provides equally good results for this task as the conventional approach, it is possible to quickly carry out analyses like the study replications shown here. We combined ad hoc IE with additional filters based on structured and unstructured data: We stratified the data by year and severity of the respective condition, and analyzed subgroups like age, comorbidities and concomitant medication. Furthermore, we used ad hoc IE to transform unstructured data from the discharge letters to structured data (e.g. systolic blood pressure groups) and extracted the daily dosage per drug on the fly.

To calculate daily medication dosages, each strength unit combination must still be queried individually. It is intended to calculate this automatically, e.g. with the use of function queries.

## Abbreviations

ADHD: Attention deficit hyperactivity disorder
AF: Atrial fibrillation
ATC: Anatomical Therapeutic Chemical classification system
BMI: Body mass index
BP: Blood pressure
CDW: Clinical data warehouse
CIS: Clinical information system
CKD: Chronic kidney disease
EHR: Electronic health record
GUI: Graphical user interface
ICD-10: International Classification of Diseases, version 10
IE: Information extraction
LVEF: Left ventricular ejection fraction
Med1: Department of Internal Medicine I
NDTI: National Disease and Therapeutic Index
NHANES: National Health and Nutrition Examination Survey
NOAC: Novel oral anticoagulants
OAC: Oral anticoagulants
OPS: Operationen- und Prozedurenschlüssel
SBP: Systolic blood pressure
T2DM: Type 2 diabetes mellitus
UKW: University Hospital of Würzburg
VKA: Vitamin K antagonist

## Footnotes

1 Extract, Transform, Load

2
http://lucene.apache.org/solr/

3
https://lucene.apache.org/core/

4
https://uima.apache.org/

5 The complete trigger set is available at: https://go.uniwue.de/padawan

6
https://www.whocc.no/atc_ddd_index/

7
http://abdata.de/datenangebot/abda-datenbank/

8
http://www.is.informatik.uni-wuerzburg.de/research_tools_download/athen/

9
http://community.i2b2.org/wiki/display/DevForum/Text+search+in+i2b2
